# How to Develop Entrepreneurial Talent More Effectively? A Comparison of Different Entrepreneurship Educational Methods

**DOI:** 10.3389/fpsyg.2021.644113

**Published:** 2021-04-15

**Authors:** Qixing Yang, Jiachun Chen, Lijun Yang, Zhenhuan Liu

**Affiliations:** ^1^Department of Management, Zhongshan Institute, University of Electronic Science and Technology of China, Zhongshan, China; ^2^Department of Computer Engineering, Dongguan Polytechnic, Dongguan, China

**Keywords:** entrepreneurial talent, entrepreneurship classroom teaching, extracurricular activities, teaching method, entrepreneurial intention

## Abstract

Recently, scholars have begun to shift focus toward the effectiveness of different teaching methods for entrepreneurship education. However, the establishment of a unified and clear standard for the division of entrepreneurship educational methods remains unfulfilled, affecting the accuracy of research conclusions. In the present study, for the first time, the aim was to divide the entrepreneurship educational method into the classroom teaching method (CTM) and the extracurricular activity method (EAM) from the perspective of competency level training. On the basis of the modified planning behavior theory, the influence of entrepreneurship education on entrepreneurial intention (EI) was explored. In the present study, 514 college students of 14 universities in China were surveyed. The results reveal that the CTM and EAM had a direct positive bearing on EI, with indirect impact exerted by attitude toward entrepreneurship (ATE) and perceived behavioral control (PBC). Although the direct effects of the two teaching methods were similar, EAM could effectively improve ATE and PBC, thereby resulting in a positive effect on EI to a greater extent. Further observations were made that the participation of research University students in CTM was significantly lower than that of applied University students, leading to lower EI. Additionally, higher EI could be attributed to the more active participation in EAM of male students than female students, while no significant difference was indicated between different majors in EI. The results are of significant reference value for promoting the reform of entrepreneurship education and improving the quality of entrepreneurship education in colleges and universities.

## Introduction

As a result of the global coronavirus disease 2019 (Covid-19) pandemic, many businesses faced difficulties or even went bankrupt. Only companies with flexible operations and the ability to adapt quickly to market changes survived. Others saw the crisis as an opportunity, being able to fulfill the new needs of the market and formulate fresh ideas therefor. The common aspect of these companies lies in their teams of entrepreneurial people who remain passionate and create a culture of constant innovation. Notably, universities play a central role in the cultivation of entrepreneurial talents (Martínez-Martínez and Ventura, 2020). Recently, scholar attention has been drawn to determining how best to teach entrepreneurship for the achievement of better results (Lackéus, 2020). Regarding the evaluation index of the impact of entrepreneurship education on results, prior research has most commonly adopted entrepreneurial intention (EI) (51%), followed by perceived feasibility (25%), entrepreneurial skills and knowledge (21%), and attitude toward entrepreneurship (ATE) (19%) (Nabi et al., 2017). As such, in the present study, EI was taken as the dependent variable to evaluate the effect and mechanism of different entrepreneurship education teaching methods on EI.

Existing studies have confirmed that the participation of college students in entrepreneurship education can enhance their knowledge of entrepreneurship, promote their perception of feasibility, and then increase their entrepreneurial willingness (Robinson and Hayes, 1991). Entrepreneurship education can promote entrepreneurship by improving the entrepreneurial skills and competence of students (Wilson et al., 2007; Kucel and Vilalta-Bufi, 2016), as well as improve their entrepreneurial willingness by changing the psychological mood and motivation of entrepreneurs and stimulating their entrepreneurial inspiration (Souitaris et al., 2007). In the process of receiving entrepreneurship education, individuals are also more likely to find team partners and obtain technical resources and even financial support, thereby enhancing EI (Souitaris et al., 2007). From the perspective of entrepreneurial attitude and motivation, entrepreneurship education has been confirmed by Fiet (2001) to have a positive impact on EI. In prior research, the majority of studies have confirmed the positive effect of entrepreneurial education on EI (Athayde, 2010), yet several studies have indicated that the effect of entrepreneurial education on EI is not significant, or even negative (Graevenitz et al., 2010; Chen et al., 2013; Lima et al., 2015). The reason for such divergence could be ascribed to the fact that many studies have included entrepreneurship education as a single variable into the research model, without considering the different effects that different teaching methods of entrepreneurship education may have.

Hence, scholars have recently begun to shift focus toward the effectiveness of different teaching methods of entrepreneurship education. Bechard and Gregoire (2005) proposed three basic archetypes: supply, demand, and capacity models. The supply model focuses on the dissemination and replication of knowledge, primarily taking the forms of lectures and reading. The demand model primarily takes the form of interactive search and simulation, with a focus on exploration, discussion, and experiment. The ability model focuses on allowing students to solve problems in real situations and primarily adopts the teaching methods of communication, discussion, and knowledge production. Based on the aforementioned framework, Nabi et al. (2017) sorted 159 studies related to entrepreneurship education from 2004 to 2016 and applied mixed models on the basis of three basic prototypes. Piperopoulos and Dimov (2014) classified entrepreneurship education courses into two types (theoretical orientation and practice orientation) while Sirelkhatim and Gangi (2015) further divided the practice-oriented teaching method into two methods (simulation entrepreneurship and practical entrepreneurship). Although these explorations are undoubtedly of considerable benefit, existing research has been more concerned with how to “teach” more effectively from the perspective of the supplier. As there is no clear and unified definition standard, there are differences in classification methods. For this reason, clarifying the classification standard of the entrepreneurship education teaching method is necessary, which is an important basis for accurately grasping the internal mechanism of entrepreneurial education on EI. In the present study, a discussion on how to learn more effectively from the perspective of the demand side (students) is provided, and the classification standard of the entrepreneurship education teaching method is proposed. This is of considerable significance, as students are the object of entrepreneurship education and the subject of entrepreneurship behavior.

In addition, scholars have started to pay due attention to the recognition of action- and experience-oriented entrepreneurship education teaching methods (Nabi et al., 2017; Neck and Corbett, 2018; Lackéus, 2020; Rosado-Cubero et al., 2021). Many colleges and universities even let students directly establish small companies to conduct dry learning, but existing studies have confirmed that this is not necessarily the most effective way (Lackéus, 2020). The aforementioned studies are based on the belief that there is a need for re-evaluation of the effectiveness of traditional classroom teaching methods (CTMs) in cultivating students' entrepreneurial ability and spirit, especially in certain developing countries, where it is not necessarily possible for every student to directly set up a company to implement the teaching method of “learning by doing.” Existing comparative studies on different teaching methods of entrepreneurship education have mostly adopted qualitative research (Nabi et al., 2017; Balan et al., 2018; Lackéus, 2020; Verduijn and Berglund, 2020), while quantitative research support remains relatively minimal. For the research object, only a certain type of major (Souitaris et al., 2007; Liñán and Chen, 2009; Mukesh et al., 2020; Rosado-Cubero et al., 2021) or a certain type of University (Ismail et al., 2018) was selected in many of the existing studies. There is a scarcity of studies comparing the influence of different entrepreneurial educational methods on EI under different majors, genders, and types of universities.

Through group interviews and questionnaires conducted for the present study, the different methods of entrepreneurship education were distinguished from the perspective of the competency level training of students, and the modified planned behavior theory was taken as the research framework to explore the influence of entrepreneurship education on the EI of college students in different majors, University types, and gender. Ultimately, owing to the present study, the quality of entrepreneurship education in Chinese universities can be improved, and a decision-making basis for EI can be further provided.

## Literature and Hypothesis

### Entrepreneurship Educational Method

As is widely accepted, entrepreneurship can be taught (Drucker, 1985; Hahn et al., 2017), but determining how to render a more effective teaching method has recently become a much discussed topic in the entrepreneurship education field (Fayolle, 2013). Sirelkhatim and Gangi (2015) summarized the entrepreneurship education teaching method into three types, namely, “about,” “for,” and “through.” The “about” entrepreneurship approach is theory-oriented, while the latter two are practice-oriented. Different from other approaches, “for” entrepreneurship usually adopts the simulation teaching method (Honig, 2004), in which a student pretends to be an entrepreneur as part of a role play. The “through” approach emphasizes the real experience of students in the entrepreneurial process and requires students to learn in the market by means of incubators (Vincett and Farlow, 2008), which is fundamentally different from the “for” approach. Piperopoulos and Dimov (2014) classified entrepreneurship courses into two types: theory-oriented courses and practice-oriented courses. In the theory-oriented entrepreneurship course, teachers are taken as the center to linearly impart entrepreneurship knowledge and usually adopt teaching methods such as classroom teaching, case studies, or inviting successful entrepreneurs to lecture, facilitating the learning of students in a passive manner. Conversely, in the practice-oriented entrepreneurship course, students learn how to start an enterprise through “learning by doing” and are considered as the center. Students can be encouraged to create a real enterprise (or at least a simulation) and build up a network with a community of entrepreneurs to conduct business. Here, students can actively learn in practice through the guidance of both teachers and entrepreneurs (Gibb, 2002). On the basis of such classification, in the research of Piperopoulos and Dimov (2014), observations were made that when students participated in practice-oriented entrepreneurship courses, their EI increased with the enhancement of entrepreneurial self-efficacy, and when they participated in theory-oriented courses, the opposite trend was exhibited. Passaro et al. (2018) compared the samples participating in the entrepreneurship theory course with another group participating in a business plan competition (BPC) and found that the participation in the theory course had no significant impact on EI. Meanwhile, participation in the entrepreneurship competition had a significantly positive impact on EI. Despite the aforementioned findings, Ismail et al. (2018) demonstrated that the “teacher-centered” CTM achieved better results in the cultural context of Malaysia. These results are obviously contradictory and pose the question of whether the theory-oriented entrepreneurship education is really invalid and whether it is appropriate to divide the teaching methods of entrepreneurship education into two types: theoretical orientation and practical orientation.

In summary, existing research has continued to adopt the thinking model of how to “teach” in a more effective way, instead of focusing on how students can “learn” more effectively. The EI of students is a vital indicator to assess the efficacy of entrepreneurship education (Nabi et al., 2017), the most important prerequisite for which being entrepreneurial self-efficacy (Chan et al., 2012). Therefore, the classification of entrepreneurship education in consideration of the competency level training will be beneficial for accurately assessing the effectiveness of the different methods of entrepreneurship education. As reported in the new version of *Education Target Taxonomy* revised by Anderson et al. (2001), the cognitive ability training of students in any educational activity can be divided into six levels from low to high: memory, comprehension, application, analysis, evaluation, and creation. The top three levels are for low-order cognitive abilities, with the bottom three being for high-order cognitive abilities. In the present study, this theory was adopted as the main basis of entrepreneurship education classification. Additionally, the classification of teaching activity formulated by Dewey (1916) was followed, where the educational method is divided into the CTM and the extracurricular activity method (EAM), as shown in Table 1. CTM is a part of the daily teaching plan, which is arranged at a certain time and place. Students can get credits through examinations. CTM focuses on training students' lower-order cognitive abilities such as memory, understanding, and application of knowledge; and common forms include entrepreneurship theory courses and experimental courses. EAM is the extracurricular activities that students participate in voluntarily. Students learn by themselves, develop innovative products or services, write business plans, and focus on training students' higher-order cognitive abilities such as analysis, evaluation, and creativity. Common forms include the entrepreneurship competition and entering the maker space. In a recent study, Mukesh et al. (2020) divided the entrepreneurship educational method into the traditional CTM and the action learning teaching method. Experimental research demonstrated that both teaching methods had a positive impact on EI, but students who participated in the action learning teaching method had higher EI than those who participated in the traditional CTM. On this basis, the following hypothesis was proposed in the present study:

**Table 1:** Classification of entrepreneurship educational methods.

**Entrepreneurship educational method**	**Classroom teaching method (CTM)**	**Extracurricular activity method (EAM)**
Cognitive training level	Focus on low-order cognitive training, such as • Memory • Understand • Application	Focus on higher-order cognitive training, such as • Analysis • Evaluation • Create
Characteristics	• Teacher centered • Student passive learning • Determine the teaching schedule and location • Individual or team work	• Student centered • Students learn actively • Arrange your own time after class • Team writing completed
Example of learning form	• Entrepreneurship theory courses • Business and management courses • Entrepreneurial simulation experiments	• Entrepreneurship competition/business plan competitions • Entrepreneurship training programs funded by the education sector • Move into an incubator or maker space

***H1:*** *From the perspective of competency level training, the entrepreneurship educational method can be divided into the CTM and the EAM*.

### Entrepreneurship Education and Entrepreneurial Intention

Referring to a self-identified belief that a person plans to create a new enterprise at some point in the future (Thompson, 2009), EI serves as the most critical predictor of entrepreneurial behavior (Krueger et al., 2000) and also a significant indicator to evaluate the effect of entrepreneurial education. In the classroom teaching process, teachers impart knowledge of entrepreneurship and business management-related theory, share the successful stories of entrepreneurs, and conduct analyses on the support of the government and the school for the entrepreneurship of college students. Students participating in the course may also meet like-minded partners, and the schools will invite external entrepreneurs to conduct lectures, so as to stimulate the EI of college students. By analyzing 42 studies, Martin et al. (2013) found that a significant positive relationship was indicated among entrepreneurship education, entrepreneurial human capital, and entrepreneurial performance. Nabi et al. (2017) reviewed 81 studies from 2004 to 2016 on the relationship between entrepreneurship education and EI, 61 of which (75%) exhibited a positive relationship between entrepreneurship education and EI. The most recent studies have also confirmed that entrepreneurship education is one of the key factors affecting EI and entrepreneurial behavior (Tung et al., 2020; Hameed et al., 2021), with several studied conducted from the perspective of China (Liu et al., 2019; Zhang et al., 2019). On this basis, the following hypothesis was proposed in the present study:

***H2a:*** *The entrepreneurship CTM has a direct and significant positive impact on the EI of college students*.

When participating in entrepreneurship competitions or applying for enterprises in the maker space and other extracurricular activities in the process, college students usually need to form teams; produce a business plan; explore opportunities that exist in the analysis of the external environment, and development and innovation of products or services; evaluate the advantages and disadvantages of their products compared with those of competitors; and collect related industrial and entrepreneurship support policies. These experiences lay the foundation for their future entrepreneurship and help stimulate EI. Tuan et al. (2019) investigated 1,600 young Vietnamese people and revealed that past experience related to entrepreneurship had a direct and significantly positive impact on EI. The research results of Passaro et al. (2018) also demonstrated that participation in BPCs had a direct and significantly positive impact thereon. On this basis, the following hypothesis was proposed in the present study:

***H2b:*** *Entrepreneurship extracurricular activities have a direct and significant positive impact on the EI of college students*.

### Entrepreneurship Education and Theory of Planned Behavior

Developed by Ajzen (1991) on the basis of theory of reasoned action (TRA) (Fishbein and Ajzen, 1975), theory of planned behavior (TPB) has been demonstrated to well explain and predict human planned behaviors in practice and has thus emerged as the most widely applied psychological theory (Kolvereid, 1996) for studying the relationship between intention and behavior. Intention is considered to be the best predictor of planned behavior (Krueger et al., 2000). Under the TPB framework, individual behavioral intention is predominantly influenced by three factors, namely, individual attitudes toward behavior, subjective norms, and perceived behavior control. Subjective norm means that the individual will measure the perceived social pressure, that is, whether people (such as parents, teachers, classmates, and friends) support the behavior, so as to assist in the decision of whether to implement said behavior. Perceived behavior control refers to the perception of an individual about the level of difficulty of realizing the benefit (Ajzen, 1991).

As entrepreneurship is a planned behavior (Bird, 1988), TPB has been extensively applied in the study thereof. Through empirical research, Krueger et al. (2000) demonstrated that the TPB theory could well predict EI. Liñán et al. (2011) developed a questionnaire on EI based on the TPB theory, reporting that ATEs and control of perceived behavior were the two most important factors to explain EI. Through quasi-experimental research, Lorz (2011) revealed that said two factors exerted significant influence on EI, while subjective norms had insignificant influence thereon. Moreover, around 1,600 young Vietnamese were surveyed by Tuan et al. (2019) to authenticate the profound impact rendered by ATE and perceived behavior control on EI. In accordance with the existing research on EI based on TPB, although the influence of ATE and perceived behavior control on EI has been unanimously recognized, divided opinions exist with regard to the impact of subjective norms. In fact, in seven out of 16 empirical studies reviewed by Ajzen (1991), observations were made that subjective norms had an insignificant contribution to the expression of different behaviors. In the aforementioned studies, only the mediating effect of attitude and perceived behavior control was considered when discussing the influence of entrepreneurship education on EI, since parents and relatives are not the direct target of entrepreneurship education in colleges, making their attitude toward student entrepreneurship hard to alter.

Nabi et al. (2017) reviewed 108 studies on the relationship between entrepreneurship education and personal psychological capital from 2004 to 2016, observing that 26 out of 32 studies related to personal attitude reported a positive influence; 28 out of 34 studies on entrepreneurial skills and knowledge reported a significant positive impact; and in 42 relevant studies, 33 reported a positive influence. Further, several recent studies corroborated the positive impact of entrepreneurship education on attitude and perceived behavior control (Vorley and Williams, 2016; Hahn et al., 2017; Ismail et al., 2018; Passaro et al., 2018; Tuan et al., 2019; Zhang et al., 2019; Mukesh et al., 2020). On this basis, the following hypotheses were proposed in the present study:

***H3a***: *CTM has a positive influence on ATE*.
***H3b***: *EAM has a positive influence on ATE*.
***H4a***: *CTM has a positive influence on perceptive behavior control*.
***H4b***: *EAM has a positive influence on perceptive behavior control*.

However, different teaching methods may have different effects on ATEs and perception-based behavior control. Based on the sample data of 88,918 students from 26 countries in the Global University Entrepreneurial Spirit Students' Survey, Hahn et al. (2017) demonstrated that the increase of entrepreneurial human capital (entrepreneurial knowledge and skills) effectuated by the high-practice-oriented entrepreneurial educational method was always higher than that from the low-practice-oriented teaching method. Through a comparative experiment, Passaro et al. (2018) confirmed that attending the theoretical course had insignificant influence on ATEs and perceived behavior control of a student, but the entrepreneurship competition could greatly affect the two variables. Also corroborated by the experimental study of Mukesh et al. (2020), students who engaged in action learning teaching had higher entrepreneurial self-efficacy than those undergoing traditional classroom teaching.

Through entrepreneurship classroom teaching and participation in entrepreneurship extracurricular activities, students can boost their sense of identity and perception of entrepreneurship feasibility, so as to increase their willingness to start a business. According to the research results of Ismail et al. (2018), the subjective perception of college students played an intermediary role in entrepreneurship education and EI. Zhang et al. (2019) selected 200 college students in Hong Kong as the research object, where a research model was established based on the planned behavior theory. Here, entrepreneurial learning was demonstrated to significantly influence the attitudes of students toward entrepreneurship and perceived behavior control, and to further affect the EI. On the basis of the aforementioned findings, the following hypotheses were proposed in the present study:

***H5:*** *ATE plays a mediating role between entrepreneurial education and EI*.

***H6:*** *Perceived behavioral control plays a mediating role between entrepreneurial education and EI*.

**Figure 1:**
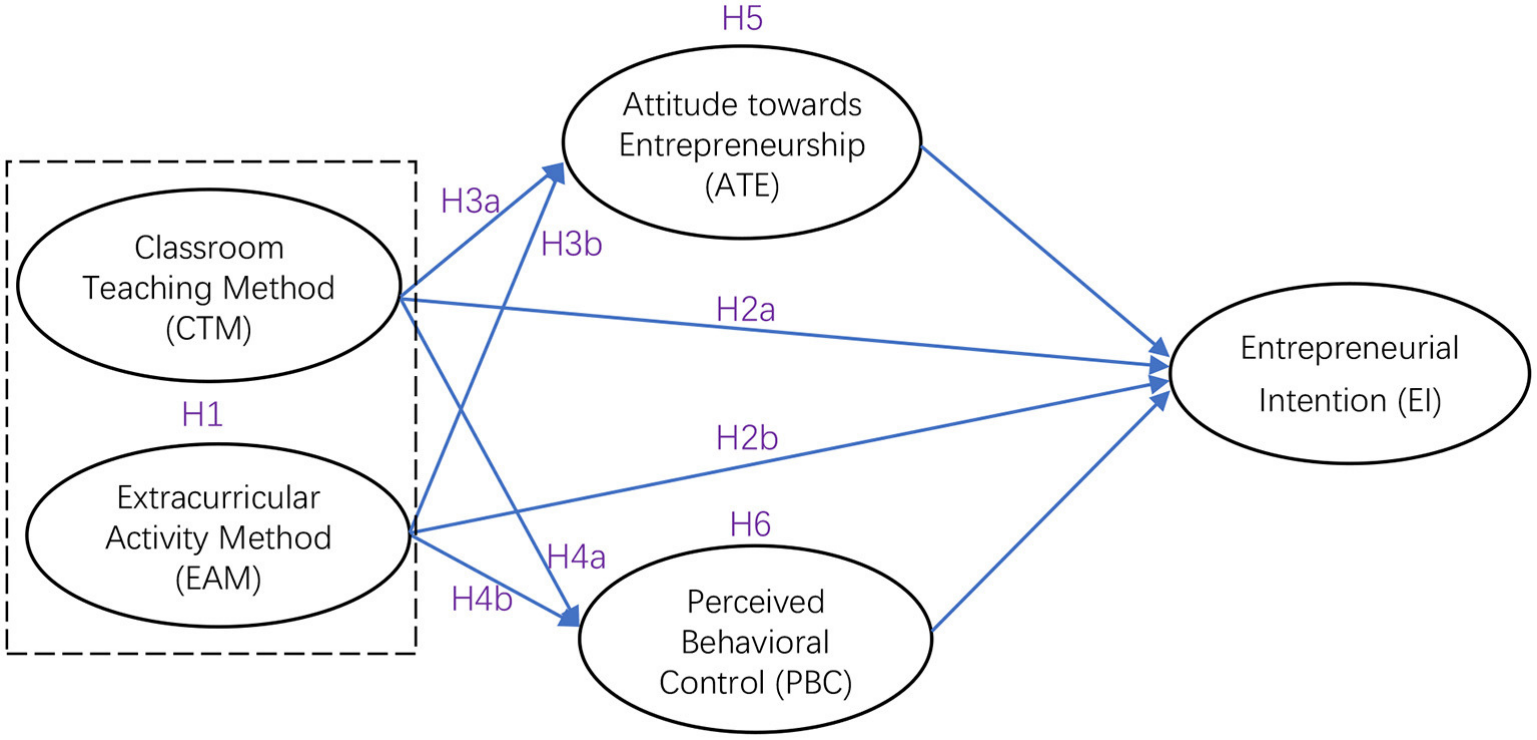
Theoretical model of the study.

Based on the above research assumptions, the theoretical model constructed for the present study is shown in Figure 1.

## Research Methods

### Variable Measurement

In the present study, to accurately investigate the involvement of Chinese college students in the entrepreneurship education teaching method, interviews were arranged for 30 senior undergraduate students prior to the investigation. The interviewees were required to list the names of entrepreneurship education activities that they were aware of. After the lists were collected, the top 80% projects most mentioned were kept in accumulative terms, which included eight projects: fundamentals of entrepreneurship education course, entrepreneurship simulation experiment, business and management course, entrepreneurship lecture of successful entrepreneurs, innovation and entrepreneurship competition, college student innovation and entrepreneurship training plan, teacher research project, and entering the maker space on campus.

By means of the scale of Krueger et al. (2000) for reference, interviewees evaluated the expected utility of starting a business by scoring four designed items, such as “starting a business can generate a sense of accomplishment.” Moreover, by means of the scale of Liñán and Chen (2009) for reference, four items were designed based on perceptual behavior control, such as “I am creative;” and four items were designed in terms of the EI, such as “I think I will start my own business in the future.” In addition, the “gender,” “type of colleges and universities,” and “major” were taken as control variables. The universities could be classified into “research universities” and “applied universities.” In the present study, universities in “Project 211” (Project 211 is a project of National Key Universities and colleges initiated in 1995 by the Ministry of Education of the People's Republic of China) were categorized as “research universities,” with the rest defined as “applied universities.” Major was divided into three types, namely, “science major,” “business major,” and “other majors.” The detailed design of the measurement items can be seen in the Appendix A.

In general, the measurement methods of entrepreneurship education can be divided into two kinds. The first involves measuring the perceived value and support of students regarding entrepreneurship education, which is a subjective evaluation and usually measured by the Likert scale (Souitaris et al., 2007; Hou et al., 2019). The second is an objective record of facts and involves asking students about their experience in entrepreneurship education. This is usually measured by asking students whether they have participated in entrepreneurship education programs (Kolvereid and Moen, 1997; Galloway and Brown, 2002; Lorz, 2011; Naia et al., 2014). Taking into account that the measurement results of the second method are more objective and the exogenicity of the variables is better, two multiple-choice questions were used in the present study, with the answers of “yes” (having a value of 1) or “no” (having a value of 0), to ask the respondents about their participation in entrepreneurship education. The other variables were investigated using the Likert seven-level scale, with “1” meaning “strongly disagree” and “7” meaning “strongly agree.”

### Sample and Data Collection

The data in the present study were acquired from the questionnaires, which were conducted by the research team and completed by students from 14 universities in China from April to June 2020. The samples covered both research and applied universities, in addition to business and science majors, thereby rendering the data relatively representative. Six hundred questionnaires were issued altogether, and 530 were recovered, with a recovery rate of 88.3%. Invalid questionnaires were eliminated, and 514 valid questionnaires were ultimately obtained, with an effective rate of 85.7%. The basic statistical characteristics of the samples are presented in Table 2.

**Table 2:** Basic sample statistical characteristics.

**Project**		**Number of samples**	**Proportion (%)**
Gender	Male	231	44.9
	Female	283	55.1
Types of colleges	Research University	158	30.7
and universities	Applied University	356	69.3
Professional	Science major	162	31.5
category	Business major	196	38.1
	Others	156	30.4
Total		514	100

## Results

### Reliability and Validity Analysis

An exploratory factor analysis and a reliability test were conducted for the scale by using SPSS 23.0 software, the results of which are shown in Table 3. The variable measurement item of the load was between 0.767 and 0.898, the scale of the overall Cronbach alpha coefficient was 0.903, the subscales of reliability coefficient were between 0.838 and 0.902, the Kaiser–Meyer–Olkin (KMO) value was between 0.745 and 0.829, and the cumulative variance contribution was between 67.458 and 77.309%. An observation can be made that the scale had a high reliability and good internal consistency. In addition, the Harman single factor test was employed to check whether common biases existed in the data. The results reveal that all items were aggregated into five factors without limiting the number of factors, the corresponding factor measurement item was exactly the same as that of the scale design, the characteristic value was >1, the cumulative variance contribution rate was 72.730%, the first factor of the total variance was explained at a rate of 39.617%, and other factors were explained at a rate of <50%. Thus, the present study could be regarded as being affected by common method biases.

**Table 3:** Results of exploratory factor analysis (*N* = 514).

**Variable**	**Measuring item**	**Load**	**Cronbach's alpha**	**KMO**	**Cumulative variance contribution (%)**
Classroom teaching method (CTM)	Q1	0.852	0.838	0.783	67.458
	Q2	0.857			
	Q3	0.806			
	Q4	0.767			
Extracurricular activity method (EAM)	Q5	0.833	0.869	0.825	71.776
	Q6	0.864			
	Q7	0.857			
	Q8	0.835			
Attitude toward entrepreneurship (ATE)	Q9	0.848	0.875	0.829	72.734
	Q10	0.858			
	Q11	0.871			
	Q12	0.833			
Perceived behavioral control (PBC)	Q13	0.859	0.861	0.745	70.611
	Q14	0.898			
	Q15	0.825			
	Q16	0.774			
Entrepreneurial intention (EI)	Q17	0.895	0.902	0.828	77.309
	Q18	0.858			
	Q19	0.881			
	Q20	0.882			

*KMO, Kaiser–Meyer–Olkin*.

The results of exploratory factor analysis indicate that the “Fundamentals of Entrepreneurship Education Course (Q1),” “Entrepreneurship Simulation Experiment (Q2),” “Business and Management Course (Q3),” and “Entrepreneurship Lecture of Successful Entrepreneurs (Q4)” were classified as the same factor, and these teaching methods all had the characteristics of classroom teaching. “Innovation and Entrepreneurship Competition (Q5),” “College Students Innovation and Entrepreneurship Training Plan (Q6),” “Teacher Research Project (Q7),” and “Entering the Maker Space on Campus (Q8)” were classified as the same factor, which was consistent with the characteristics of the extracurricular activity teaching method, and preliminary verification of H1's classification of the entrepreneurship education teaching method. In order to further verify the hypothesis, in-depth interviews were conducted with 30 students, who were asked to evaluate the effects of these eight teaching activities on the competency level training of student. The results exhibit that there were several differences among students only in Q2. All respondents agreed that the “Entrepreneurship Simulation Experiment” could train understanding and application of entrepreneurial knowledge, which is low-order cognitive ability training. At the same time, 11 students (36.67%) believed that the “Entrepreneurship Simulation Experiment” could also train the higher-order abilities of problem analysis and problem solving. Overall, respondents tended to believe that the “simulation of entrepreneurship” focused on training lower-order cognitive abilities, believing that the teaching method was still teacher-led despite being practical. The interviewees reached a consensus on the competency level training corresponding to other teaching methods, believing that Q1, Q3, and Q4 focused on the training of lower-order cognitive abilities, while Q5–Q8 focused on the training of higher-order cognitive abilities, thereby verifying H1.

For the purpose of testing the convergence validity and discriminant validity of the scale, the verification factor analysis of the survey results was conducted with AMOS 26.0 software. The results are provided in Table 4, and an observation can be made that the goodness-of-fit index (GFI) of the five-factor model was good [χ^2^/df = 3.306, comparative fit index (CFI) = 0.940, GFI = 0.903, incremental fit index (IFI) = 0.940, normed fit index (NFI) = 0.917, root mean square error of approximation (RMSEA) = 0.067]. The correlation coefficient between all variables (*p* < 0.001) was significantly correlated. The average variance extracted (AVE) values were >0.5, the variables and the variables' AVE square root were greater than the correlation coefficient of this variable with other variables, and the composite reliability (CR) value was >0.8. The scale had good convergent validity and discriminant validity.

**Table 4:** Descriptive statistics, correlation coefficient, and AVE and CR values of variables (*N* = 514).

**Variable**	**CTM**	**EAM**	**ATE**	**PBC**	**EI**	**AVE**	**CR**
CTM	0.756					0.572	0.841
EAM	0.719^***^	0.790				0.625	0.869
ATE	0.372^***^	0.377^***^	0.799			0.638	0.876
PBC	0.475^***^	0.543^***^	0.446^***^	0.784		0.614	0.861
EI	0.481^***^	0.506^***^	0.397^***^	0.573^***^	0.835	0.698	0.902
The average	0.609	0.552	5.425	5.036	4.546		
The standard deviation	0.400	0.421	0.991	0.963	1.237		

*** *means P < 0.001, and the data on the diagonal are the square root of AVE for each variable*.

*CTM, classroom teaching method; EAM, extracurricular activity method; ATE, attitude toward entrepreneurship; PBC, perceived behavioral control; EI, entrepreneurial intention; AVE, average variance extracted; CR, composite reliability*.

### Difference Test

A difference test was conducted through independent sample T testing and one-way ANOVA, and the results are listed in Table 5. An observation can be made that there was no significant difference between male and female students in CTM participation level. However, a significant difference existed between the EAM participation level and EI, in that male students' participation level and EI were considerably higher than those of female students. Additionally, there was no significant difference between research and application-oriented universities in terms of participation in EAM, yet CTM participation and EI participation were different. The CTM participation of students in application-oriented universities was notably higher than that of research-oriented universities, which was the same for EI. Finally, there was no significant difference in CTM, EAM participation, and EI among students of different majors.

**Table 5:** Difference test results.

**Sample characteristics**	**Category**	**Entrepreneurship classroom teaching**			**Entrepreneurship extracurricular activities**			**Entrepreneurial intention**		
		**The mean**	**The standard deviation**	**Difference significance**	**The mean**	**The standard deviation**	**Difference significance**	**The mean**	**The standard deviation**	**Difference significance**
Gender	Male	0.632	0.402	0.248	0.609	0.403	^**^	4.701	1.260	^*^
	Female	0.591	0.397		0.505	0.430		4.420	1.205	
Types of colleges and universities	Research-oriented University	0.555	0.398	^*^	0.547	0.405	0.867	4.337	1.325	^*^
	Application-oriented University	0.633	0.399		0.554	0.428		4.639	1.186	
Major	Science	0.596	0.415	0.096	0.596	0.409	0.199	4.681	1.242	0.247
	Business	0.656	0.379		0.515	0.435		4.490	1.163	
	Others	0.566	0.405		0.553	0.414		4.478	1.316	

* means p < 0.05;

** *p < 0.01*.

### Hypothesis Testing

**Figure 2:**
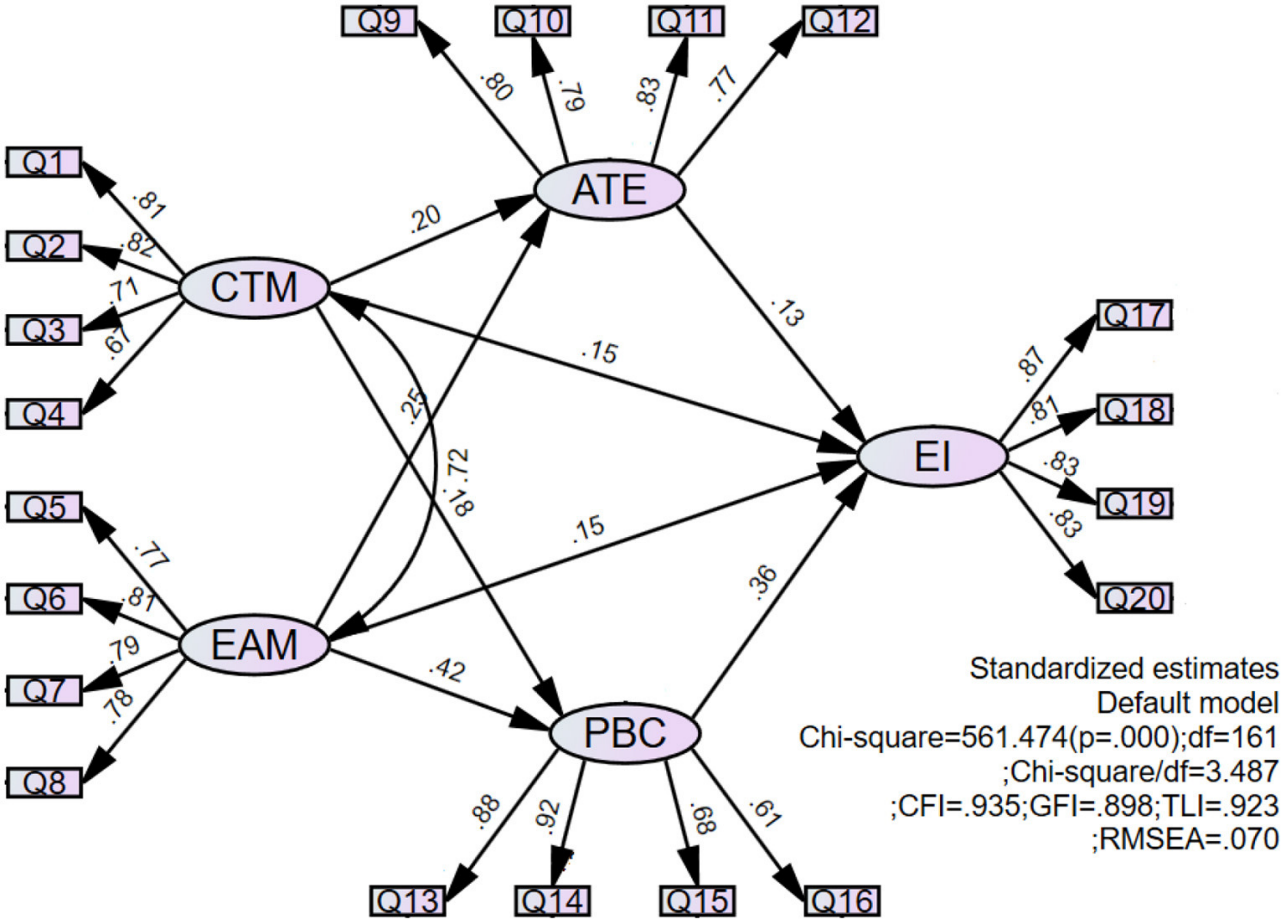
Model test results.

To test the hypothesis of the theoretical model shown in Figure 1, AMOS 26.0 software was employed in the present study. As shown in Figure 2 and Table 6, the GFI of the model was good [χ^2^/DF = 3.487, CFI = 0.935, GFI = 0.898, Tucker–Lewis index (TLI) = 0.923, RMSEA = 0.070]. The results illustrate that CTM and EAM had a direct and significant positive impact on EI, with the standardized coefficients being 0.154 (*P* < 0.05) and 0.152 (*P* < 0.05), respectively, thereby verifying H2a and H2b. The results of the mediation effect analysis are provided in Table 7. The total effect of CTM on EI was 0.245, while that of EAM on EI was 0.339. The bias-corrected 95% confidence interval did not include 0, and the total effect of EAM on EI was greater than that of CTM.

**Table 6:** Results of path analysis and hypothesis testing.

**The path**	**Nonstandardized path coefficient**	**Normalized path coefficient**	**SE**	**CR**	**Significance level**
CTM → EI	0.557	0.154	0.248	2.249	^*^
EAM → EI	0.467	0.152	0.222	2.102	^*^
CTM → ATE	0.573	0.199	0.226	2.539	^*^
CTM → PBC	0.343	0.177	0.139	2.464	^*^
EAM → ATE	0.617	0.252	0.191	3.233	^**^
EAM → PBC	0.698	0.423	0.125	5.576	^***^
ATE → EI	0.162	0.129	0.057	2.835	^**^
PBC → EI	0.679	0.364	0.102	6.626	^***^

* *means P < 0.05*,

** means P < 0.01, and

*** *means P < 0.001*.

*CR, composite reliability; CTM, classroom teaching method; EI, entrepreneurial intention; EAM, extracurricular activity method; ATE, attitude toward entrepreneurship; PBC, perceived behavioral control*.

**Table 7:** Results of mediation effect analysis.

**The path**		**Effect of value**	**Bias-corrected 95% CI**		**The total effect**	**Significance level**
		**The lower limit**	**Ceiling**	**The proportion**		
CTM–EI	The total effect	0.245	0.088	0.401	100%	^**^
	CTM–ATE–EI	0.026	0.004	0.065	10.61%	^*^
	CTM–PBC–EI	0.064	0.013	0.138	26.12%	^*^
	Total indirect effect	0.090	0.024	0.173	36.73%	^**^
	Direct effect	0.154	0.006	0.294	63.27%	^*^
EAM–EI	The total effect	0.339	0.186	0.495	100%	^**^
	EAM–ATE–EI	0.033	0.004	0.078	9.73%	^*^
	EAM–PBC–EI	0.154	0.078	0.25	45.43%	^**^
	Total indirect effect	0.187	0.106	0.286	55.16%	^**^
	Direct effect	0.152	0.005	0.312	44.84%	^*^

* *means P < 0.05*,

** *means P < 0.01*.

*CTM, classroom teaching method; EI, entrepreneurial intention; ATE, attitude toward entrepreneurship; EAM, extracurricular activity method; PBC, perceived behavioral control*.

As presented in Table 6, both CTM (β = 0.199, *P* < 0.05) and EAM (β = 0.252, *P* < 0.01) had significantly positive effects on ATE, thereby verifying H3a and H3b. Both CTM (β = 0.177, *P* < 0.05) and EAM (β = 0.423, *P* < 0.001) had significantly positive effects on perceived behavioral control (PBC), thereby verifying H4a and H4b. Further, in terms of the standardized path coefficient, EAM had a more positive impact on ATE and PBC compared with CTM. Notably, CTM had a greater impact on ATE than PBC, while EAM had a smaller impact on ATE than PBC, indicating that the effect of CTM participation was more reflected in the change of students' attitudes, while the effect of EAM participation was more reflected in the improvement of their ability.

As can be observed in Table 7, the direct effect and indirect effect of EI in CTM were 0.154 and 0.090, respectively, accounting for 63.27 and 36.73% of the total. In the indirect effect, the mediation effect value of ATE was 0.026, accounting for 10.61% of the total effect; and the intermediary effect value of PBC was 0.064, accounting for 26.12%. The bias-corrected 95% confidence interval of every path did not contain 0. Thus, a conclusion could be drawn that the participation of college students in CTM had both a direct impact and an indirect impact on EI by changing ATE and PBC, with the direct impact being stronger than the indirect impact. ATE and PBC played a partial mediating role between CTM and EI.

In the total effect of extracurricular activities on the EI, the direct effect was 0.152, accounting for 44.84% of the total effect, while the indirect effect was 0.187, accounting for 55.16%. In the indirect effect, the mediation effect value of ATE was 0.033, accounting for 9.73% of the total, while the mediation effect value of PBC was 0.154, accounting for 45.43%. As shown in Table 7, the bias-corrected 95% confidence interval of every path did not contain 0. A conclusion could be drawn that the participation of college students in EAM had both direct and indirect effects on EI, and ATE and PBC played a mediating role between EAM and EI. As a result, H5 and H6 were verified. Notably, different from CTM, EAM affected EI more indirectly by changing ATE and PBC, and the indirect influence was greater than the direct influence.

## Discussion

### Research Conclusion

In the present study, the competency level training of students was taken as the classification standard, and entrepreneurship education was divided into CTM and EAM teaching methods. With the competency level training as the independent variable and EI as the dependent variable, a research model was built based on the TPB theory. By employing questionnaire surveys and AMOS26.0 software, empirical research was conducted, the results of the hypothesis test show the following:

1. The participation of college students in both CTM and EAM could create a direct and significantly positive impact on EI, and there was merely a slight difference between the direct effect of CTM (=0.154, *P* < 0.05) and EAM (=0.152, *P* < 0.05) on EI.
2. In addition to direct influence on EI, CTM, and EAM also indirectly affected EI through control of ATEs and perceived behavior. The indirect effect values of CTM and EAM on EI were 0.090 (*P* < 0.01) and 0.187 (*P* < 0.01), respectively, and the bias-corrected 95% confidence interval did not include 0. Generally, the direct influence of CTM on EI was stronger than the indirect influence thereof, while the direct influence of EAM was weaker than the indirect influence thereof. Overall, the total effect of EAM on EI (0.339) was larger than CTM (0.245).
3. The results of path analysis reveal that the direct impact of perceived behavior control (=0.364, *P* < 0.001) was the largest among the four direct factors affecting EI, with the other three variables being CTM (=0.154, *P* < 0.05), EAM (=0.152, *P* < 0.05), and ATE (=0.129, *P* < 0.01). The impact of EAM on perceived behavior control (=0.423, *P* < 0.001) was significantly greater than CTM (=0.177, *P* < 0.05), thereby demonstrating that college students' perception of their ability to innovate, analyze, and solve problems was the most significant factor affecting their EI, and that participation in EAM could improve their perception of this ability more effectively than CTM.
4. In combining the results of difference analysis and hypothesis testing, an observation can be made that because male students participated in EAM more actively than female students, the EI of male students was significantly higher than that of female students. The EI of application-oriented college students was significantly higher than that of research-oriented college students, which could potentially be attributed to the fact that application-oriented college students were more involved in CTM, as there was no significant difference between these two types of college students in terms of participation in EAM. There was no significant difference in CTM and EAM participation and EI among students of different majors.

### Theoretical Significance and Practical Enlightenment

#### Theoretical Significance

Firstly, the present study facilitates re-understanding of the role of the traditional CTM in entrepreneurship education. In the study of entrepreneurial education, teaching methods have seen numerous iterations over the years, including teacher-centered (1980's), process-centered (1990's), context-centered (2000's), and learner-centered (2010's). Recently, scholar attention has shifted toward action-oriented teaching models (Lahn and Erikson, 2016), constructivist learning methods (Robinson et al., 2016), design-based thinking and lean start-up (Harms, 2015; Daniel, 2016), and the business model canvas (Osterwalder and Pigneur, 2010). The belief of the present author is that too much focus is being centered on the action- and experience-oriented teaching methods, with a study confirming that for students who start small, entrepreneurial learning is not necessarily the most effective method (Lackéus, 2020). Further, the cost of implementing this learning method is considerably high and not necessarily suitable in every education context. Returning to the fundamental goal of entrepreneurship education, although hotly debated (Neck and Corbett, 2018), entrepreneurship education as a teaching method is gaining general recognition, through which students can learn entrepreneurial knowledge, skills, and entrepreneurship and is not solely for the purpose of explaining how to start a new company (Hägg and Gabrielsson, 2020). Thus, the central goal of the present study is to bring awareness to the fact that the traditional CTM also conforms to the basic goal of entrepreneurship education, so as to improve college students' knowledge, ability, and entrepreneurship. The direct influence of CTM on EI is even greater than that of EAM, and entrepreneurship education should not ignore the role of CTM.

Secondly, in the present study, a clear and novel classification standard for teaching methods of entrepreneurship education is proposed. Prior research has not provided a unified and unambiguous definition standard for the classification of entrepreneurship education, resulting in different conclusions on the internal mechanism of entrepreneurial education affecting EI (Galvão et al., 2018; Ismail et al., 2018; Passaro et al., 2018). The present study is the first to propose taking the perspective of the competency level training of students. Here, the entrepreneurship educational method was divided into CTM and EAM, which is of considerable theoretical significance for further understanding of the connotation of entrepreneurship education. Table 1 provides guidance on the classification of entrepreneurship educational methods to avoid confusion. For instance, the majority of previous studies have classified entrepreneurship simulation courses as practice-oriented, in the same category as entrepreneurial competition and incubator methods (Piperopoulos and Dimov, 2014; Sirelkhatim and Gangi, 2015). However, these courses are actually substantially different. In simulation courses, students participate in the class according to the environmental parameters set by the teacher, with a focus on training the ability of students to understand and apply existing knowledge, which belongs to the category of low-order cognitive ability. Yet entrepreneurship competitions or activities in the maker space require students to form teams and determine the direction of the project. Here, students can experience the real environment and analyze and evaluate the market demand, products, or service innovation, with a focus on the training of higher-order cognitive abilities. Thus, the entrepreneurial self-efficacy influence of students should be different, and the quantitative studies in the present study also confirm this. The classification method provided in Table 1 is more conducive to identifying the differences in the influence of different entrepreneurship education and teaching modes on entrepreneurial self-efficacy and EI.

Thirdly, the present study provides quantitative support for the effect evaluation of entrepreneurship education teaching methods. Assessing the impact of teaching methods on students is becoming increasingly important (Ball, 2013). Balan et al. (2018) argued that in the process of entrepreneurship education, student participation is considerably important and applied team-based learning teaching methods to entrepreneurship education, so as to improve the student participation in entrepreneurship education, by using a qualitative abductive research method. The study revealed that the participation of the students subjected to the teaching method was relatively high, and several suggestions were also provided for improving the teaching method. Verduijn and Berglund (2020) proposed the introduction of critical pedagogy into the classroom teaching of entrepreneurship education. Here, in the process of deconstructing and reconfiguring the start-up, teachers treat students as co-learners, arouse their curiosity, and encourage co-creation. However, Verduijn and Berglund did not provide information about the actual effect of this teaching method with quantitative support. As for the comparison of the effects of different teaching methods, a recent study by Lackéus (2020) divided the teaching methods of entrepreneurship education into “Idea and Artifact-Creation Pedagogy” (IACP), “Value-Creation Pedagogy” (VaCP), and “Venture Creation Pedagogy” (VeCP), combining qualitative and quantitative research and using 10,953 survey data of 1,048 participants. A conclusion was drawn that there were significant differences in the effect of the three educational methods; VaCP had a considerably positive effect on improving students' entrepreneurial ability, stimulating students' entrepreneurial passion, and improving students' participation in entrepreneurship course learning and was significantly higher than the other two teaching methods. The influence of IACP on students' entrepreneurship was minimal, while VeCP did not allow the students to have a more in-depth study curriculum content, knowledge, and skills. The aforementioned study was based on the theory of motivation (Fiske, 2008), in which IACP is attributed to hedonistic motives and VeCP is attributed to egoistic motives. Notably, only VaCP is oriented to create value for others, whereby students can create motivation and passion for entrepreneurial learning through events involving “interaction with the outside world” and “creating value for others.” As such, Lackéus (2020) is of the belief that the most effective entrepreneurship education does not require students to directly start a business but only requires them to adopt a simulation entrepreneurship teaching method that is oriented to create value for others and society. An observation can be made that the existing research has focused more on the effect assessment of a single teaching method or different experience-based teaching methods and has mostly adopted qualitative research, while there is a scarcity of quantitative comparison. In the present study, taking CTM and EAM as exogenous variables and based on the simplified TPB theory, a theoretical model was constructed and empirically tests were conducted, before the different effects of different teaching methods of entrepreneurship education on the EI of college students were compared. The present study can assist researchers in more accurately grasping the internal mechanism of entrepreneurial education on EI, and provides quantitative research support for the effect evaluation of entrepreneurship education teaching methods.

#### Practical Inspiration

The present study provides enlightenment on how colleges and universities should conduct entrepreneurship education to better improve the EI of college students.

First of all, CTM and EAM both have a significantly positive influence on EI, and the total effect of the latter is greater than that of the former. Hence, in addition to traditional classroom teaching, universities should encourage students to participate more in business competitions, scientific research projects, application of innovative entrepreneurship training programs, and activities in a maker space, incubator, or other extracurricular activities, which can more effectively promote their EI.

Secondly, regardless of CTM or EAM, colleges and universities should attach considerable value to the training of the innovation, problem analysis, and problem-solving abilities of students, so as to improve their perception of the feasibility of entrepreneurial behavior, since perceived behavior control is the biggest and most direct factor affecting EI.

Thirdly, predominantly due to the significant differences in CTM participation, the EI of students in research-oriented universities is significantly lower than that of students in application-oriented universities in China. Thus, research-oriented universities should improve the EI of their students by increasing the proportion of entrepreneurship courses in the talent training program, so that as many students as possible have access to “what is entrepreneurship,” “the positive significance of entrepreneurship,” and “the basic laws and principles of entrepreneurship.” In doing so, students' positive evaluation of entrepreneurship and self-perception of entrepreneurial behavior control can be enhanced. Fourthly, encouraging female students to participate more in EAM is an effective way to improve their EI.

## Research Limitation

The limitations of the present study include that the data obtained by the questionnaire surveys were cross-sectional and lacking longitudinal comparison of data before and after the students participated in entrepreneurship education. Moreover, there was also a shortage of lateral comparative data of the control group and experimental group, and the research samples were primarily from undergraduate students without information of students of other academic degrees or those have graduated. Hence, research methods and sample selection can be further improved in the future.

## Data Availability Statement

The raw data supporting the conclusions of this article will be made available by the authors, without undue reservation.

## Ethics Statement

Ethical review and approval was not required for the study on human participants in accordance with the local legislation and institutional requirements. Written informed consent for participation was not required for this study in accordance with the national legislation and the institutional requirements.

## Author Contributions

QY determined the research theme, research framework, questionnaire design, data analysis method, and was responsible for the finalization of the paper. JC was responsible for literature collation, questionnaire implementation, and draft writing. LY was responsible for the writing of research hypothesis, data analysis, and result discussion. ZL was responsible for the collection, collation of literature, and the collation and analysis of data. All authors contributed to the article and approved the submitted version.

## Conflict of Interest

The authors declare that the research was conducted in the absence of any commercial or financial relationships that could be construed as a potential conflict of interest.

## Appendix A. Questionnaire Items of the Study

**Table:**

**I. Have you participated in any of the following entrepreneurship education activities?**		
Q1: Fundamentals of entrepreneurship education course	□ Yes	□ No
Q2: Entrepreneurship simulation experiment	□ Yes	□ No
Q3: Business and management course	□ Yes	□ No
Q4: Successful entrepreneur lecture on entrepreneurship	□ Yes	□ No
Q5: Innovation and entrepreneurship competition	□ Yes	□ No
Q6: College student innovation and entrepreneurship training plan	□ Yes	□ No
Q7: Teacher research project	□ Yes	□ No
Q8: Entering the maker space on campus	□ Yes	□ No

**Table:**

**II. Indicate your level of agreement with the following statements, “1” meaning “strongly disagree” and “7” meaning “strongly agree”**	
**Items**	**Options**
Q9: Starting a business can accumulate capital and wealth	1 2 3 4 5 6 7
Q10: Starting a business can bring you a sense of accomplishment	1 2 3 4 5 6 7
Q11: Starting a business can elevate your social status	1 2 3 4 5 6 7
Q12: Starting a business can contribute more to society	1 2 3 4 5 6 7
Q13: I am creative	1 2 3 4 5 6 7
Q14: I have the ability to innovate	1 2 3 4 5 6 7
Q15: I trust my ability to deal with problems	1 2 3 4 5 6 7
Q16: I can always solve a problem if I try my best	1 2 3 4 5 6 7
Q17: I think I will start a business in the future	1 2 3 4 5 6 7
Q18: If I have the opportunity and freedom to make decisions, I will choose to start my own business	1 2 3 4 5 6 7
Q19: Regardless of practical difficulties, I would still choose to start my own business	1 2 3 4 5 6 7
Q20: I have a good chance of starting my own business in the next 5 years	1 2 3 4 5 6 7

**Table:**

**III. Sample Characteristics**			
21. Gender:	□ Male	□ Female	
22. Type of colleges and universities:	□ Project 211	□General University	□ Academy
23. Major:	□ Science major	□ Business major	□ Other majors
